# A Study of the Automated Eddy Current Detection of Cracks in Steel Plates

**DOI:** 10.1007/s10921-019-0647-9

**Published:** 2019-12-28

**Authors:** Ehsan Mohseni, Hamid Habibzadeh Boukani, Demartonne Ramos França, Martin Viens

**Affiliations:** 1 Center for Ultrasonic Engineering (CUE), Department of Electronics and Electrical Engineering, Technology and Innovation Centre, University of Strathclyde, 99 George Street, Glasgow, G1 1RD UK; 2 Département de génie mécanique, L’École de technologie supérieure, 1100 Rue Notre-Dame O, Montréal, QC H3C 1 K3 Canada; 3 Zetec Inc, 875 Boulevard Charest O, Québec, QC G1N 2C9 Canada; 4 John Abbott College, 21275 Lakeshore Dr, Sainte-Anne-de-Bellevue, QC H9X 3L9 Canada

**Keywords:** Non-destructive testing (NDT), Eddy current testing (ECT), Split-D reflection differential probe, Eddy current noise, Probability of detection (POD), NDT reliability

## Abstract

Applying life estimation approaches to determine in-service life of structures and plan the inspection schedules accordingly are becoming acceptable safety design procedures in aerospace. However, these design systems shall be fed with reliable parameters related to material properties, loading conditions and defect characteristics. In this context, the role of non-destructive (NDT) testing reliability is of high importance in detecting and sizing defects. Eddy current test (ECT) is an electromagnetic NDT method frequently used to inspect tiny surface fatigue cracks in sensitive industries. Owing to the new advances in robotic technologies, there is a trend to integrate the ECT into automated systems to perform NDT inspections more efficiently. In fact, ECT can be effectively automated as to increase the coverage, repeatability and scanning speed. The reliability of ECT scanning, however, should be thoroughly investigated and compared to conventional modes of applications to obtain a better understanding of the advantages and shortcomings related to this technique. In this contribution, a series of manual and automated ECT tests are carried out on a set of samples using a split-D reflection differential surface probe. The study investigates the level of noise recorded in each technique and discuss its dependency on different parameters, such as surface roughness and frequency. Afterwards, a description of the effect of crack orientation on ECT signal amplitude is provided through experimental tests and finite element simulations. Finally, the reliability of each ECT technique is investigated by means of probability of detection (POD) curves. POD parameters are then extracted and compared to examine the effect of scanning index, frequency and automation on detection reliability.

## Introduction

Fatigue failure is the most important source of damage in systems subjected to cyclic loads. In the aerospace industry, this phenomenon is frequently observed as the dynamic nature of stresses during flight, takeoff and landing promotes the nucleation of micro-cracks and the propagation of existing short cracks. In order to make decisions on the continuation of operation and also on the maintenance intervals, risk assessment programs have been introduced [1]. In this framework, probabilistic physical models are used to estimate the remaining fatigue life of in-service components. These models are mostly developed based on the damage tolerance approach, which requires several input parameters including flaw characteristics, material properties and loading conditions [2]. Optimal definition of these input parameters considering their uncertainty would lead to more accurate remaining life estimation; thus, the maintenance intervals would be set based on more realistic results, and unexpected failure could be avoided [3]. Concerning the flaw characterization, non-destructive testing (NDT) methods are the most available and practical means. The uncertainty of flaw characterization is largely influenced by the NDT method, inspection device, test conditions, component under test and inspector [4]. To address this issue, the reliability and capability of NDT methods in flaw characterization should be properly assessed. Probability of detection (POD) has been developed as a measure to quantify such reliability. Based on POD results it would be possible to make a better decision about the largest flaw which may be missed by a given NDT method [5]. Beside POD as the main reliability metric, probability of false indication (POFI) should also be investigated in the context of NDT reliability. Most of the programmed inspection plans employ POD curves along with POFI as an advantageous means to determine the inspection intervals in some critical safety fields, in particular aerospace industries [6, 7]. It is always of high importance to select the NDT technique and related equipment objectively to efficiently detect flaws within structures. The appropriate selection of technique and inspection apparatus as well as the test parameters can increase the POD and assure the integrity and reliability of the in-service component over its expected life time [8].

Considering its simplicity of operation and effectiveness, eddy current testing (ECT) is one of the NDT methods widely used for detecting and sizing fatigue cracks in the aerospace industry [9]. ECT, which is based on electromagnetic principles, is one of the preferred methods for the inspection of surface discontinuities in electrically conducting materials [10]. Since the nucleation sites for fatigue cracks are mainly located on the surface of materials, reflection differential split-D ECT probes can be a good option for the detection of such cracks. The placement of D-shaped receiver coils in the housing and their differential configuration provide a small footprint and high signal to noise ratio. This could be translated into high detection sensitivity for surface cracks, reducing undesirable noises caused by the probe’s lift-off and tilt [11, 12]. The advantages of using this type of probe configuration become more pronounced when inspecting ferromagnetic materials (e.g., martensitic steels), since the ECT signals detected from these materials could be very noisy. Different studies have investigated the performance of split-D probes through model-based approaches [12–17], while some others have tried to perform model-based inversion based on the flaws scanned by the probes [18–20].

Knowing that ECT is often used in the modern aerospace industry, where the quality of the inspection method plays a critical role in the public safety, it is crucial to investigate the reliability of the ECT method through POD studies. Like any other NDT technique, ECT signals are always accompanied with variability in tests that could impact the POD [21]. Therefore, POD studies on ECT have become increasingly important in recent years. Rosell et al. presented a comparative study on automated and manual scans of surface cracks using an absolute ECT probe [22]. Moreover, a series of studies regarding POD of ECT inspections of bolt-hole were conducted by Krause et al. [23–25]. They generated PODs based on the inspection results for fatigue cracks and EDM (electrical discharge machined) notches located in bolt holes of bi-layer 7075-T6 aluminum sheets. Their inspections were performed using rotary split-D ECT probes, and the effect of different calibration schemes, such as two-point calibration, on the obtained POD were investigated.

The present research aims to investigate, for the first time, the reliability of both manual and automated (encoded) scans for detecting surface fatigue cracks in a set of flat AISI 410 steel samples using a split-D probe. To this end, the signal response POD is considered as the quantitative tool for this comparison study, where only the most influential parameters, namely the test frequency, the crack orientation, the index of automated raster scan and the inspector are considered. The methods used herein for POD data analysis are in accordance with the MIL Handbook 1823A standards [5] and are using the mh1823 POD algorithms package, which is available online [26]. Even though this study does not cover all the usual round robin of test parameters, the small matrix of laboratory tests conducted herein shows to be fairly conclusive as a comparative study.

During the analysis of the signal amplitude as a function of the relative orientation between the scan and crack lines, it is observed that for each specific orientation there is a threshold crack length above which the changes of eddy current signal amplitude become less than 5%. Hence, one could speculate that the signal amplitude is becoming almost insensitive to the crack length. Unfortunately, the crack length interval used in the experiments is limited. In order to expand the extent of this study to larger crack sizes, and thus gain a better insight into the experimental observation, finite element modelling (FEM) is employed. For this purpose, a FEM is prepared for a split-D probe interacting with samples containing semi-elliptical notches representative of fatigue cracks. Subsequently, the effect of notch length and orientation on the signal amplitude is analysed through FEM simulations.

The paper structure is organized in the following manner. The variables treated as the source of variability in ECT response for both automated and manual ECT scans are presented in section two. Section three provides the details of experimental procedures and calibration system. In section four, noise analysis for each technique is presented, and the dependency of the noise on different test parameters is investigated. The effect of crack orientation on the signal amplitude is studied experimentally and numerically in sections five and six. The reliability studies concerning both techniques through POD curves are discussed in section seven and the study is concluded in section eight.

## ECT POD Variables

Obtaining variable responses by repeated inspection of a flaw with a fixed size is a proof of a lack of reproducibility for a given NDT technique. Scanning responses depend on many factors including the material properties, characteristics of the flaw, equipment set-up, inspector skills and environmental factors. Accordingly, for a specific flaw size, a distribution of signal amplitudes could be obtained, which could be used further to generate a POD curve using statistical analysis.

An intuitive insight into the parameters affecting signals must be achieved when generating a POD curve for a certain ECT application. Special care should be taken to include the influential parameters while insignificant ones could sometimes be neglected. Table 1 lists several parameters that may contribute to the flaw response variability [22].

**Table 1:** List of parameters causing variations in ECT signals during automated and hand scans

Hand scans	Automated tests
Inspector	Index and speed of scan
Squaring collar	Vibrations and associated noise
Environmental conditions	
Calibration	
ECT equipment and probe	
Test frequency	
Gain and electrical noise	
Probe orientation (differential probe)	
Probe’s tilt and lift off	
Sample’s surface conditions (curvatures, roughness and contaminations)	
Material properties (conductivity and permeability)	
Crack geometry (shape, opening, profile, length and depth) and orientation	
Signal acquisition and feature extraction for POD analysis	

Even though some of the parameters presented in Table 1, such as environmental conditions, may contribute to POD results, their contribution is negligible compared to some influential ones and might not be included in a POD study. Since the same ECT instrument and probe are used in both manual and automated scans, they are not considered as a possible source of variation in this study. During the tests, the probe is positioned randomly on the sample’s surface leading to possible variations in the relative angle that the scan direction makes with the orientation of milling tool marks on the surface. Although effort is put in keeping the perpendicularity of the probe relative to the surface, using a bell-shaped squaring collar in manual scans and a micrometric alignment fixture in automated scans, small tilt angles may still remain. The small tilt variations along with the effect of tool marks orientation are considered as contribution to lift-off. The speed of automated scans, 10 mm/s, is low relative to the data acquisition sampling rate of 10,000 samples/s; therefore, the effect of scanning speed is disregarded. In view of these assumptions, it is decided to only consider a subset of influential parameters in this study. A list of these parameters is summarized in Table 2.

**Table 2:** List of parameters examined in this work for monitoring their effect on the distribution of ECT response

Hand scans	Automated tests
Inspector	Scanning index
Test frequency	
Crack orientation	

## Experimental Procedure

The specimens used in this study were provided by an aircraft engine manufacturing company. They are made of martensitic AISI 410 steel with a 114.3 mm × 25.4 mm × 6.35 mm dimensions. Pristine samples were mixed with others containing artificially induced fatigue cracks. Specimens are machined from three steel plates. Fatigue cracks are grown out of a small starter EDM notch using cyclically loaded three-point bending tests. Cracks are grown to predetermined lengths to cover a useful range. Then, the top and bottom surfaces of the samples are machined off so that the starter notches and fixture marks are removed. Destructive tests performed on a subset of these samples revealed that the depth (*D*) of fatigue cracks is correlated to their length (*L*) through the linear regression given in Eq. 1 with a coefficient of determination *R*^*2*^ = 0.984. A total number of 21 samples are used in this small-scaled POD study, where 5 of these samples are blank and the rest are defective. The length and the depth of the smallest crack are measured as 0.76 mm and 0.22 mm while these dimensions are 2.95 mm and 1.01 mm for the largest crack.

1 $$ D{ = } - 0.006\; + \;0.347\;L $$

A Nortec 500S eddy current device along with a reflection split-D differential surface probe with a frequency band in the range of 500 kHz to 3 MHz are selected for the inspections. The experimental tests are performed in two separate stages: (a) first, all samples including defective and undefective are inspected manually; (b) second, an automated raster scan is conducted on the entire set of samples. In both stages, horizontal and vertical gains of the Nortec device are fine tuned to fit the largest captured signal within 80% of both screen height and width at each test frequency. Accordingly, the response of the largest fatigue crack included in the study (2.95 mm in length and 1.01 mm in depth) is used for calibration purpose. To do so, this crack is scanned in several passes perpendicular to its length and only the maximum impedance responses of the Fig. 8 signal of the differential probe were used to set the gains, so the signal extremities matches 80% of the screen height and width. Although, the signal of a defect scanned by a split-D probe is not always symmetrical due to the differences that may occur during manufacturing of the D-shaped cores and coils, but the shape is less important in a POD study as long as the peak-to-peak distance is correctly calibrated on the screen. Such an approach has been suggested by Krause et al. [24, 27] because the use of larger cracks for the purpose of calibration decreases the variability caused by the calibration that different inspectors perform. Besides, the signal obtained from these cracks is higher in amplitude giving rise to the best available signal to noise ratio.

Manual ECT scans are performed by three qualified inspectors. During the manual scans, a bell-shaped squaring collar is attached to the probe to maintain the perpendicularity of the differential probe to the sample’s surface. In addition, a Teflon tape with thickness of 50 μm is used on the probe’s tip. This helps to reduce the friction between the probe and the scanning surface, making it easier for the probe to glide over the surface during the inspection process.

**Fig. 1:**
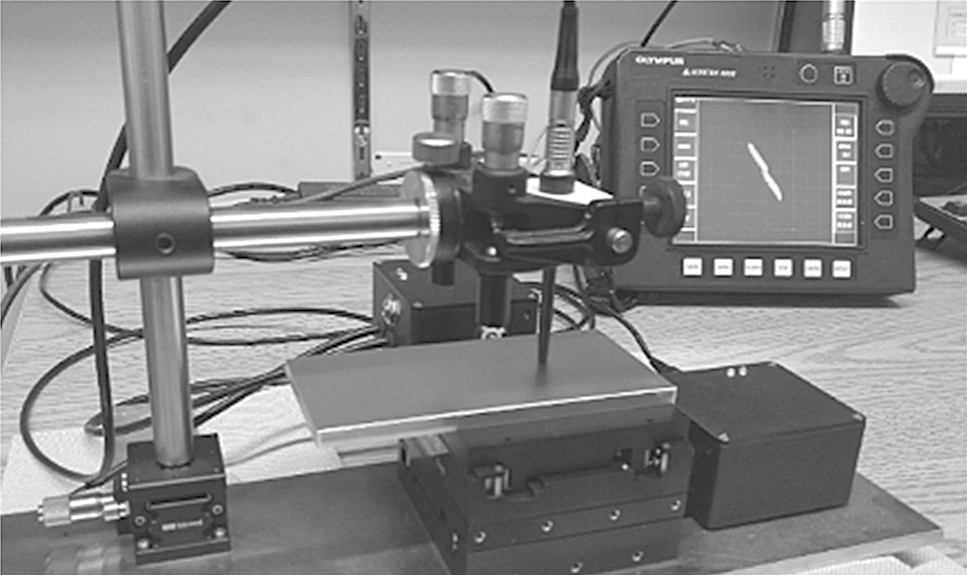
Experimental setup for performing automated ECT scans

For automated scanning, a motorized X–Y table is utilized to perform a raster scan on the samples. The ECT probe is clamped inside an alignment holder, allowing gimbal and swivel micrometric rotation about the axis of a mounting post. The lift-off is controlled through a micrometric Z-stage and set to 30 ± 10 µm to avoid friction between the probe’s tip and the sample’s surface, thus preventing the probe from flexing. It is notable that the probe’s lift-off is set once at the scan’s start point; therefore, the error in lift-off adjustment is mainly dictated by the limited precision of the micrometric knob on the Z-stage whereas the surface waviness is measured, LEXT OLS4000 surface laser profiler by Olympus Inc. to be 10% of this error. The experimental setup for the automated scans is shown in Fig. 1. Before starting each scan, the probe is positioned arbitrarily on the sample’s surface to randomize the relative position of the probe and cracks, if there is any. Subsequently, three scans with indexes of 2.5 mm, 1.25 mm and 0.5 mm are conducted for each sample while keeping the same probe position at the start of the scan.

**Fig. 2:**
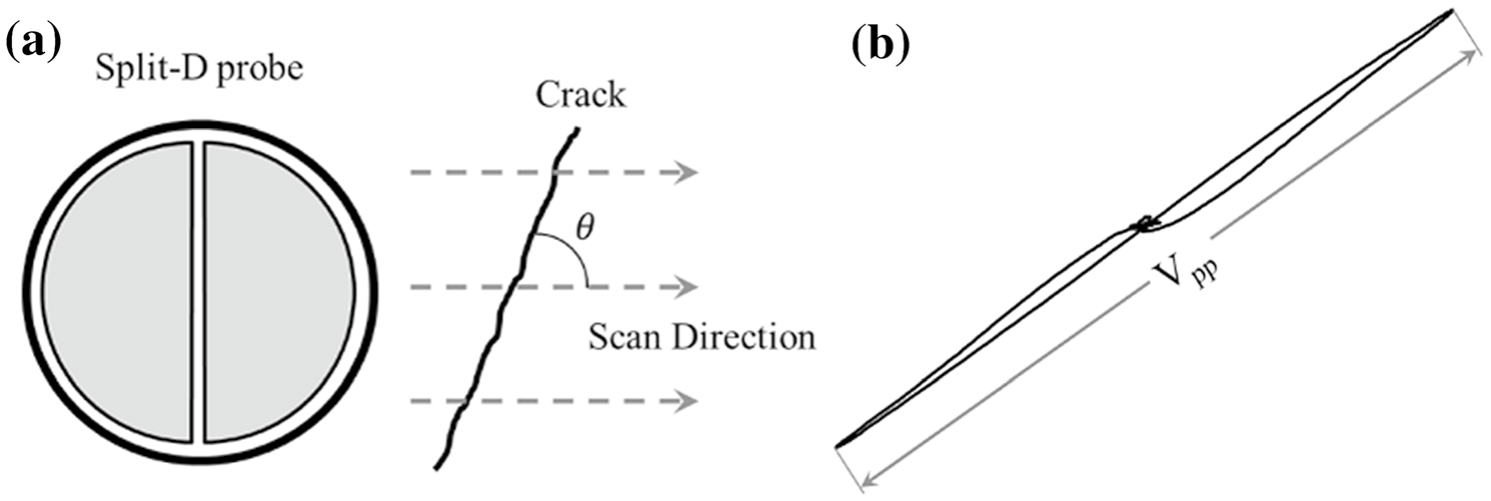
**a** relative orientation represented by the angle *Ɵ*, which is defined by the crack line and scan direction, and **b** peak-to-peak amplitude of an 8-shape ECT signal

A solvent cleaner is applied on the sample’s surface before starting the inspections to remove any potential contamination. All samples are tested at three test frequencies of 500 kHz, 750 kHz and 1 MHz, and orientation angles of 0°, 45° and 90° (refer to *θ* in Fig. 2a). The signals captured by the Nortec 500S device are recorded and transferred to a computer through a data acquisition card for further processing. The Fig. 8 impedance trajectories are plotted from the recorded data of horizontal and vertical axes, and then are post-processed in MATLAB to find the peak-to-peak amplitude values for all indications (refer to *V*_*pp*_ in Fig. 2b).

## Noise Treatment

As mentioned in Sect. 2, sample surface conditions, such as roughness, contribute to ECT noise. In addition, for manual scanning, surface roughness may induce probe wobble and vibration due to handling and pressure variation during scan. For automated scanning, undesired effect, such as non-parallelism between the sample surface and the scan plan, is another contributor to noise. There is finally a fundamental noise coming from electrical circuitry.

Electrical noise has first been measured by nulling the Nortec device while the probe was held in the air. The signal acquire thereafter has been processed in MATLAB^®^ to calculate the root mean square (RMS) variation of the probe impedance, $Z_{rms}$. Such a calculation is based on Eq. 2 where $X(t)$ and $R(t)$ are respectively the inductive reactance and the resistance of the probe as a function of time. It has later been observed that less than 10% of the noise level recorded during the scans is attributed to electrical noise.

2 $$ Z(t) = \sqrt {X(t)^{2} + R(t)^{2} } ,\quad Z_{rms} = \sqrt {\frac{1}{{(t_{2} - t_{1} )}}\smallint\limits_{{t_{1} }}^{{t_{2} }} {\left[ {Z(t)} \right]^{2} dt} } $$

**Fig. 3:**
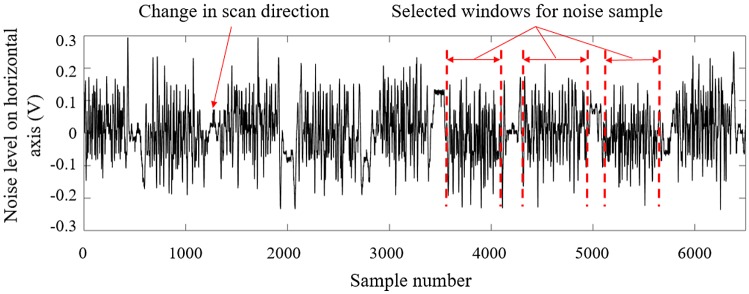
Typical noise recorded during manual scan and the windows selected for processing

To calculate the noise level associated to sample scanning, the signals acquired from undefective samples are imported to MATLAB^®^. As depicted in Fig. 3, a typical noise signal is interrupted by intervals during which scanning direction is reversed. In order to disregard these intervals, noise samples are taken within windows that exclude these inconsistent signals. A moving average over each of these windows is calculated and subtracted from the sample’s response to compensate for the effects of the probe’s tilt and lift-off during each pass of the scan. This pre-processing operation is carried out in an attempt to isolate noise due to surface conditions from the one induced by non-ideal probe handling. Afterwards, the RMS impedance is calculated according to Eq. 2 for each window, and the values of 10 windows are averaged to yield the noise level for one scanning condition.

**Fig. 4:**
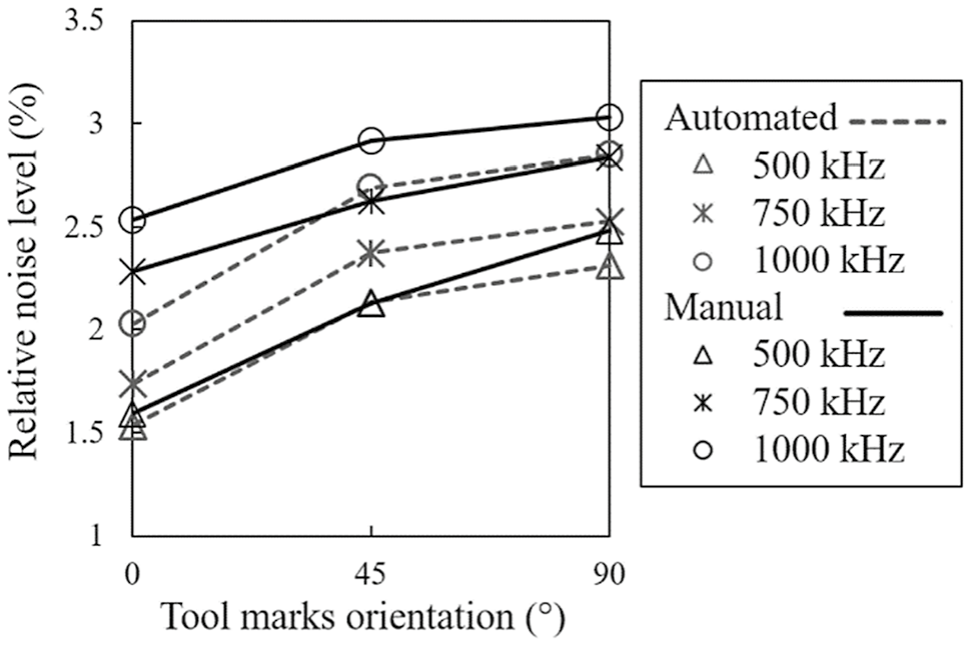
Variations of the percentage of noise level normalized by the calibration signal amplitude as angle between the probe scan direction and machining tool marks orientation changes. Results are presented for 3 frequencies for both manual and automated scans

Because the eddy current device gains differ with operating frequencies and scanning techniques (manual or automated), noise level shall be analysed in accordance with these settings. So, in all cases, noise level has been normalized with respect to the calibration signal amplitude. The noise variations against the orientation of the machining tool marks with respect to scanning direction are plotted in Fig. 4. From this plot, it can be found that noise of the manual scan shows higher values at all testing conditions as compared to the automated scans. The lift-off introduced by the Teflon tape during manual scans is 50 μm, which is larger than the one used in automated scans. The higher lift-off distance results in weaker coupling between the probe’s electromagnetic field and sample. To compensate for such an effect, gains on each axis should be increased during calibration, and this fact alone elevates the level of noise in manual scans. Moreover, in comparison to automated scans, manual scans are affected by some other sources of noise, including the non-uniformity of hand pressure during the scanning process and fluctuations imposed by the surfaces’ irregularities, which raise the overall received noise on the eddy current signal.

**Fig. 5:**
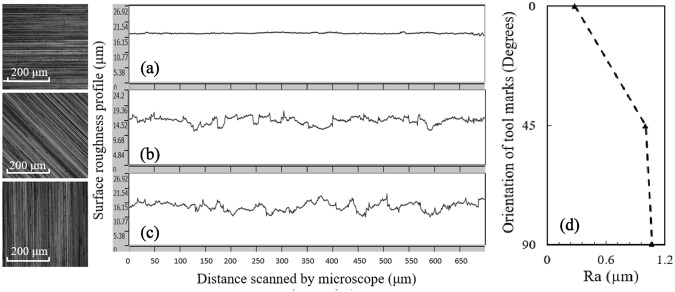
Optical laser microscope images and samples of the surface roughness profile for **a** 0°, **b** 45°, and **c** 90° angles that the probe’s scan direction makes with the orientation of tool marks on surface. **d** orientation of tool marks versus $R_{a}$

Both manual and automated noise levels get larger as the relative angle between the probe and machining tool path direction (*i.e.*, tool marks orientation) increases from 0° to 90°. In the same fashion, the arithmetic mean deviation of the assessed profile ($R_{a}$) increases as the surface tool marks orientation changes from 0° to 90°, according to Fig. 5. Therefore, as we look at the variations of the noise level with the tool marks orientation in Fig. 4, the trend of variations closely follows the one observed for $R_{a}$ against the tool marks orientation in both techniques. However, the noise growth rate of automated scans more accurately resembles the $R_{a}$ variation trend. This can be explained by observing that the scan direction relative to the tool marks orientation is more accurate in the automated scan than in the manual. Furthermore, the additional noise superimposed by other sources in manual scans, as mentioned previously, could contribute to the slight deviation of the slope of manual noise variations from that of $R_{a}$.

As another influential parameter, an increase in frequency would also intensify the noise levels in both techniques. Variation of the noise level as the frequency is increased from 500 kHz to 1 MHz seems to be quasi-linear in automated scans, whereas the trend is moderately different in manual scans. As it is observed in Fig. 4, the noise associated to the manual scan grows relatively faster as the frequency changes from 500 kHz to 750 kHz when compared to the growth rate in the 750 kHz and 1 MHz interval. Since the material under investigation is ferromagnetic, the skin depth of eddy currents at 750 kHz approaches that of the surface’s roughness. Therefore, further increase in frequency has a weaker effect on the noise level.

## Numerical Modeling for Orientation Study

To investigate the effect of crack orientation on the signal amplitude of the split-D probe, a 3-dimensional model for the assembly of split-D probe and sample is prepared in Comsol Multiphysics. The scan of surface notches with different lengths at three orientations of 0°, 45° and 90° is carried out to simulate the experimental tests. The frequency of 1 MHz is chosen for the model-based study. It shall be noted that in these simulations, fatigue cracks are replaced by semi-elliptical notches as they can fairly represent the shape of fatigue cracks. Also, the tight opening of 20 μm is considered for the notches to provide a better estimation of fatigue crack signals. According to the notch opening study presented in [11], however, the maximum signal amplitude becomes less dependent on notch opening as the notch gets larger in dimensions. The models for 0° and 90° orientations are cut in half across the symmetry plane to save simulation run time. On the other hand, the scans with 45° orientation have no symmetry plane, and simulations for this orientation shall be conducted with a full-scaled model. The details regarding the Comsol model for the assembly of the probe and sample along with the selected physics and solvers are discussed in [11]. The mesh is slightly changed as compared to that study, since the material is ferromagnetic and requires a finer boundary layered mesh. Therefore, the entire geometry is meshed using second order tetrahedral elements and 8 boundary layers, each having a thickness of 20 μm starting from the surface of the sample. The thickness of each layer is almost equal to the skin depth of eddy currents in the steel sample. In addition, very fine elements (with the size of almost one standard penetration depth of eddy currents in the sample) are used for meshing the notch walls where a high concentration of eddy currents is expected due to perturbation. The rest of the model is freely meshed by selecting a very low growth rate within the domain volumes.

**Fig. 6:**
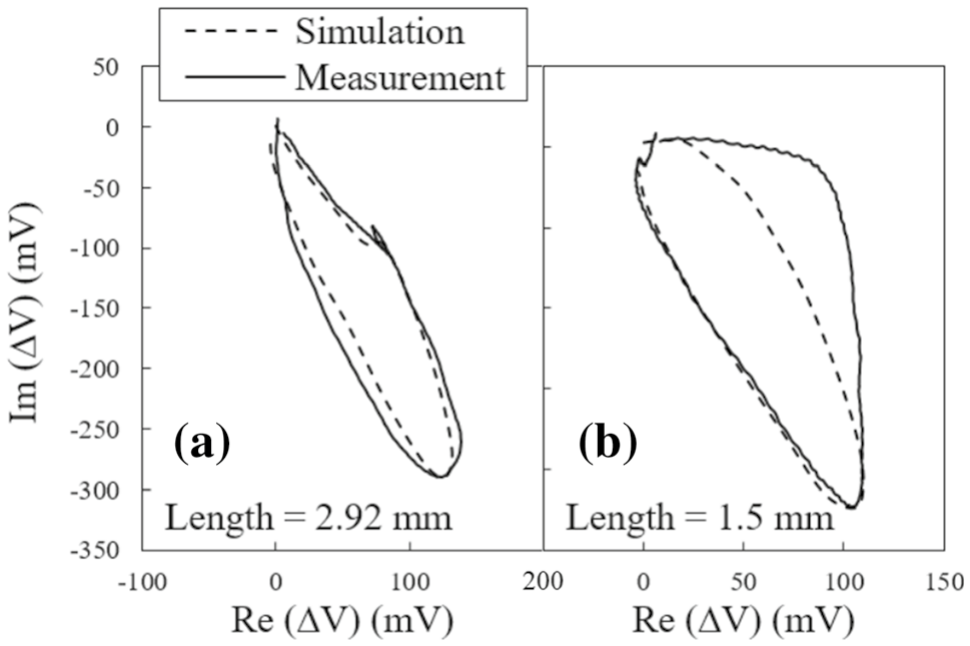
Comparison between the measured signal from a crack and simulated signal for a notch for a **a** 2.92 mm and **b** 1.5 mm long crack/notch oriented at 0°

Before conducting the study, the validity of the model is examined by comparing the simulation results with the experimental test results for two cracks with 2.92 mm and 1.50 mm in length, both oriented at 0° relative to the scan direction. These results are plotted together in Fig. 6a and b, respectively. The simulated and experimental signals are quite similar in terms of amplitude levels; however, there is a discrepancy between the shape of the simulated and experimental signals for a 1.5 mm long crack. This could be attributed to the difference between the geometry of the simulated straight notches, which possess parallel side walls, and the zigzag shaped fatigue cracks. Another possible source of error may be that the probe is not scanned over the exact position at which the simulation is carried out. Furthermore, the deviation of the notch opening and profile from the real geometry of a fatigue crack, as small as 1.5 mm in length, can contribute to the signal shape discrepancies as well. It is worthy to note that the important signal feature required for validating our model-based case study is the maximum amplitude, for which the matching between the simulation and experimental results is quite acceptable. Following the model verification, the notch length is varied with incremental steps of 0.5 mm within the size intervals presented in Table 3, and the simulations are performed for all the notch sizes listed in the table.

**Table 3:** The length interval studied through modelling for each specific orientation

Orientation	Min. length (mm)	Max. length (mm)
0°	0.5	3.0
45°	1.5	4.5
90°	2.0	6.0

## Effect of Crack Orientation on Signal Amplitude

**Fig. 7:**
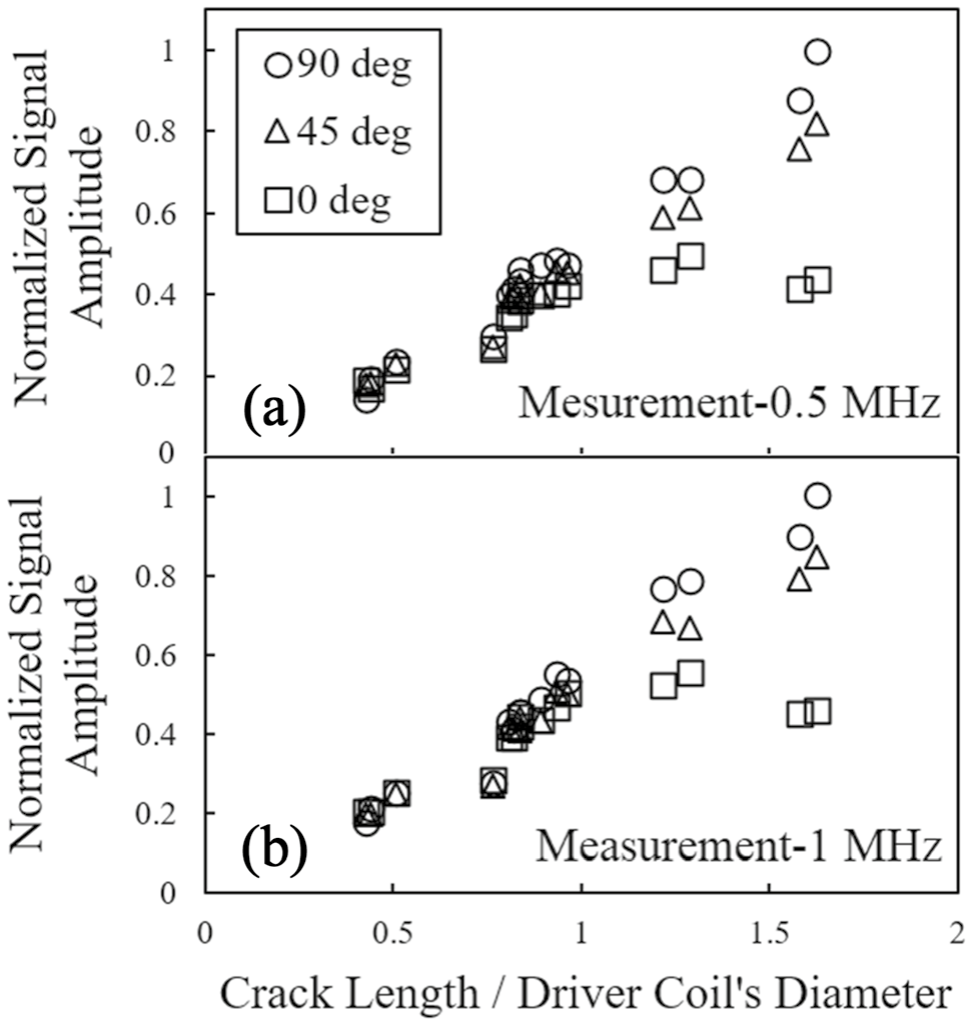
Normalized signal amplitudes versus *L/D* ratio measured using a reflection differential split-D probe for orientations of 0°, 45° and 90° at frequencies of **a** 0.5 MHz and **b** 1 MHz

Signal amplitudes obtained at frequencies of 500 kHz and 1 MHz from automated scans are normalized and plotted versus the crack length to driver coil’s diameter ratio (*L/D*) for the three orientations in Fig. 7. At each crack length, the corresponding signal amplitude is normalized by the signal amplitude of the calibration crack oriented at 90°. According to the results of the 0° orientation, and up to a given *L/D*, the signal amplitude increases with the *L/D*. However, the amplitude remains almost constant beyond that value. Since such a threshold is dependent of the probe’s geometry, it is better to describe this value as a function of *L/D*, where the drive coil’s diameter is 1.8 mm. Referring back to Fig. 7, the plateau for 0° starts once the *L/D* ratio approaches the unity. For the other two orientations, the existence of that critical value could not be observed in the measurement results, meaning that the normalized amplitude keeps increasing as the *L/D* grows. Comparing the two graphs, it can be seen that increasing the frequency to 1 MHz would slightly augment the normalized amplitude for each *L/D*. In addition, each of these graphs shows that for the *L/D* values exceeding the unity, the 90° orientation generates higher signal amplitudes as compared to the other two orientations. A plausible explanation could be that at 90° orientation, the perturbation caused by eddy currents is more severe, and the resulting field distribution as well as the differential impedance are affected more significantly. However, for flaws with *L/D* approximately less than unity, the normalized signal amplitude is almost independent of the orientation. This is important in terms of probability of detection, since for flaw sizes lower than the drive coil’s diameter, the flaw orientation does not have an influential effect on the probability of detection, whereas the flaw orientation becomes a principal parameter for larger sizes. (This behaviour will be also observed later herein in the distribution of the $\hat{a}$ vs. $a$ plot depicted in Fig. 10a. In that plot, it is evident that the dispersion of amplitude values for ECT signals recorded for cracks of different orientations is significantly less at crack sizes smaller than 2 mm.)

According to the results shown in Fig. 7, for each orientation the signal amplitude is associated with some deviations from a smooth correlation with *L/D*. Even though the fatigue cracks are produced under controlled conditions, those deviations could be explained by observing that the shape and opening of the cracks might vary, yielding to a certain level of deviation in the amplitude-length correlation.

**Fig. 8:**
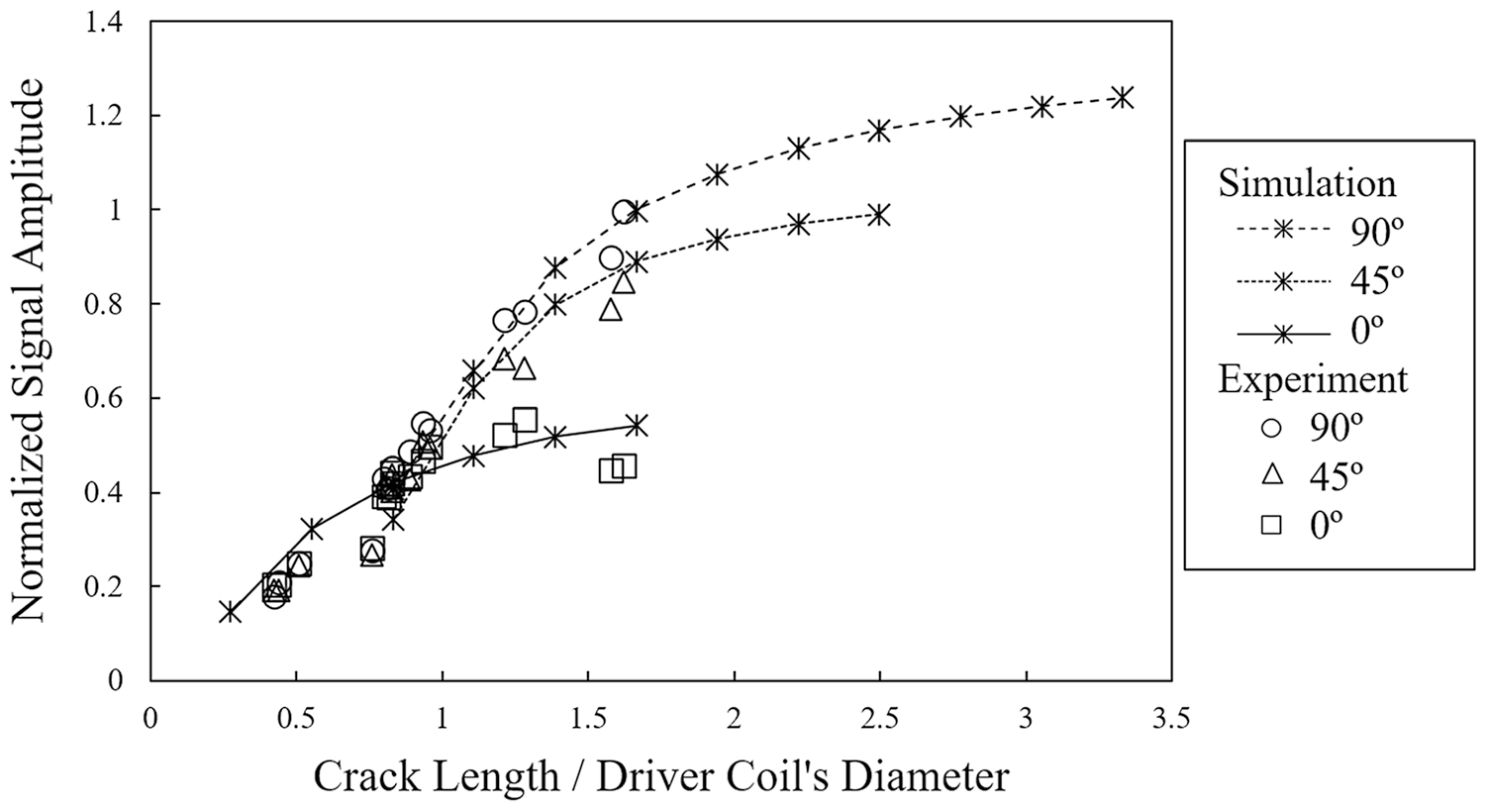
Simulated and measured normalized signal amplitudes versus *L/D* ratio for orientations of 0°, 45° and 90° at 1 MHz

Since the thresholds for the 45° and 90° orientations do not fall within the experimental *L/D* intervals presented here, the *L/D* ratios beyond these intervals are explored with the aid of FEM simulations. The simulation results are compared (superimposed) with the experimental measurements, performed at the same frequency, and presented in Fig. 8. According to this figure, there is also a critical *L/D* for each of 45° and 90° orientations, after which the signal amplitude remains almost unchanged. Figure 8 shows that the critical *L/D* could not be determined for the 45° and 90° orientations by the measurements, since they were conducted on a limited crack length’s interval having a maximum *L/D* value of 1.62, whereas the simulation results cover larger flaw lengths with a maximum *L/D* of 3.33. The threshold *L/D* ratios are found to be 1.38 and 1.66 for the orientations of 45° and 90°, respectively. The simulation results also shows the same trend as that of the experiments for the 0° orientation. It is noticed that as the orientation angle increases, the normalized amplitudes versus the *L/D* grow with a higher rate until they reach the threshold *L/D* value. All these observations confirm that the orientation has a very significant effect on the signal amplitudes after a given *L/D*. Therefore, for cracks with a *L/D* ratio higher than unity, the sizing of the crack will be associated with larger errors if the probe does not intercept the crack in a favorable direction.

**Fig. 9:**
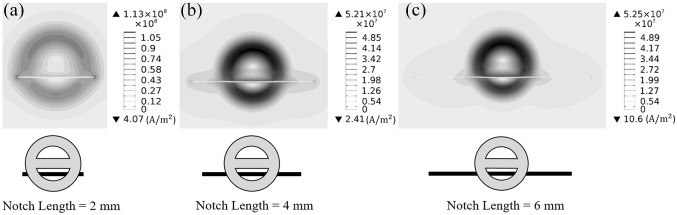
Contours of current density norm distribution on the surface of a sample. The probe center is located 0.3 mm away from the notch center. Notches with lengths of **a** 2 mm, **b** 4 mm, and **c** 6 mm are investigated

For the 90° orientation, the distribution of current density on the surface of the steel sample is demonstrated in Fig. 9a. As shown in this figure, for a 2 mm notch with *L/D* almost equal to unity, the probe’s amplitude becomes a maximum when the notch is positioned 0.3 mm away from the center axis of the probe. In this situation, the notch disturbs the high density eddy current loops, which are formed adjacent to the driver coil on the surface. Therefore, the concentration of surface currents increases in the extremities of the notch length, since currents flow from sides to bypass it. Figure 9b demonstrates that further increase of the notch length from 2 to 4 mm continues to change the distribution of eddy currents on the surface. Accordingly, the probe’s differential impedance increases, as the larger notch acts as a stronger barrier, and perturbs the current flow more significantly. However, when the notch length goes beyond the threshold value of 1.66 for *L/D*, as it has already been discussed, the changes in the electromagnetic field distribution (and thus the current density distribution) on the surface become trivial. This can be verified by comparing Fig. 9b and c. In fact, it is evident that a change from 4 to 6 mm in the notch length results in a very small influence on the surface current density and its distribution. The impact of the notch length on the current density distribution is more pronounced when comparing Fig. 9a and b. To explain this, the fact that the probe’s impedance is related to the distribution of eddy currents in the sample should be considered. Accordingly, there is not any remarkable impedance changes for crack lengths larger than the threshold *L/D* value, since this distribution does not change noticeably. In other words, for notches longer than 3 mm, the flow of eddy currents from the notch sides becomes insignificant. As a result, the contribution of the length to impedance variations fades away, since the preferred path of current flow would be different. It is also notable that because of the small skin depths (well sub 0.1 mm), cracks of 2 mm or more go far deeper than the skin depth, and the length is the main contributor to the slow growth of signal amplitude.

## POD of Automated and Manual Tests

A signal response POD analysis, according to the procedure provided in MIL-HDBK-1823, is performed on the $\hat{a}$ vs. $a$ data acquired from both manual and automated scans [5]. As the first step in this analysis, it is observed that a linear relationship can be established between log (*â*) and log (*a*) as presented in Eq. 3. In this equation, $\beta_{0}$ and $\beta_{1}$ are the regression line’s coefficients, and $\tau_{1}$ is the random error, which is assumed to have a normal distribution with a mean value equal to zero. The standard deviation of $\tau_{1}$ is presented by $\sigma_{\tau }$. After finding the regression parameters, the POD of size $a$ can be calculated through Eq. 4, where $\varPhi$ stands for cumulative log–normal function and $\hat{a}_{dec}$ is normally determined based on the noise distribution and a POFI value that is required to be achieved [7, 28].

3 $$ {\text{Log (}}\hat{a} )\; { = }\;\beta_{0} + \beta_{1} {\text{Log (}}a ) { + }\tau_{1} $$

4 $$ {\text{POD (}}a )\;{ = }\;\varPhi \left\{ {\frac{{{\text{Log}}(a) - \left[ {{\text{Log}}(\hat{a}_{dec} ) - \beta_{0} } \right]/\beta_{1} }}{{\sigma_{\tau } /\beta_{1} }}} \right\} $$

**Fig. 10:**
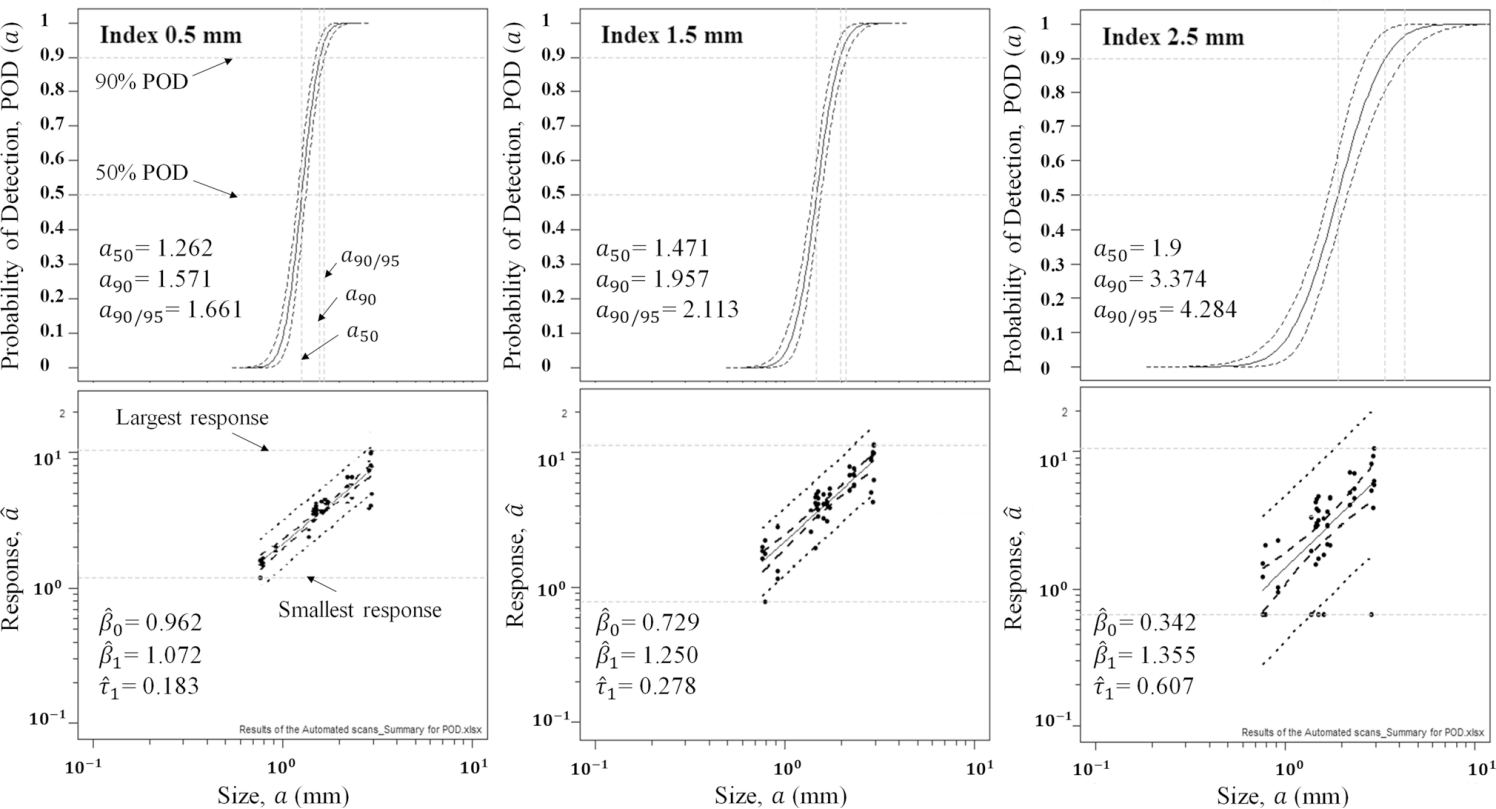
Regression lines found for $\hat{a}$ vs. $a$ data and related POD curves plotted for automated scans with indexes of **a** 0.5 mm, **b** 1.25 mm and **c** 2.5 mm

$\hat{a}_{dec}$ is taken as 30% of the signal amplitude of the calibration crack. Figure 10a demonstrates the regression line correlating $\hat{a}$ and $a$ data, as well as the corresponding POD curve plotted for the scan index of 0.5 mm in automated inspection at 500 kHz.

Figure 10b and c also present the curves extracted for scan indexes of 1.25 mm and 2.5 mm, respectively. As expected, the comparison between these curves reveals that $a_{90/95}$ improves as the scan index becomes smaller. Moving from scan index of 2.5 mm towards 1.25 mm, $a_{90/95}$ improves by 50%, and this improvement continues by 21% as the index reduces from 1.25 to 0.5 mm. This noticeable difference between $a_{90/95}$ values of 2.5 mm and 1.25 mm scan occurs because any scan index larger than the driver coil’s diameter increases the chance of missing cracks. This is the case especially when the crack is oriented 0° with respect to the scan line. As can be seen in the $\hat{a}$ vs. $a$ data plot presented in Fig. 10c, there are 4 crack sizes at which the crack is missed. On the other hand, in the case of using 1.25 mm scan index, all the cracks are detected based on the results shown in Fig. 10b. However, as compared to the index of 0.5 mm, there is a lower probability that the probe passes over the crack center at the index of 1.25 mm; therefore, the recorded signal response at this index can be lower for some crack sizes. It is well known by the ECT practitioners that choosing scan indexes smaller than the probe’s diameter improves the detection probability. The observations presented in Fig. 10 support this idea. Similar results have also been suggested in a POD study presented for automated inspections using an absolute probe [22]. Automated scans POD parameters acquired at different test frequencies and scan indexes are listed in Table 4.

**Table 4:** POD parameters for automated scans

Frequency (kHz)	Scan Index (mm)	$a_{50}$ (mm)	$a_{90}$ (mm)	$a_{90/95}$ (mm)
1000	0.50	1.3	1.6	1.7
	1.25	1.5	2.0	2.2
	2.50	2.0	3.6	4.7
750	0.50	1.3	1.6	1.7
	1.25	1.5	2.0	2.1
	2.50	1.9	3.3	4.1
500	0.50	1.3	1.6	1.7
	1.25	1.5	2.0	2.1
	2.50	1.9	3.4	4.3

According to Table 4, there is not basis to suggest that there is any change in $a_{90/95}$ as frequency changes. However, the table suggests that $a_{90/95}$ becomes smaller as the scan index is reduced, regardless of the selected frequency.

Table 5 provides the POD parameters for the manual scans performed at three different frequencies. Similar to the case of automated scan, it is not possible to draw any conclusions regarding the effect of frequency on $a_{90/95}$ of manual scans.

**Table 5:** POD parameters concerning the manual scans at different frequencies

Frequency (kHz)	$a_{50}$ (mm)	$a_{90}$ (mm)	$a_{90/95}$ (mm)
1000	1.3	1.6	1.7
750	1.2	1.5	1.6
500	1.3	1.7	1.7

Comparing Tables 4 and 5, it is evident that the value of $a_{90/95}$ for frequency 750 kHz is lower in manual scans, implying a better POD. $a_{90/95}$ values at frequency of 1 MHz show an equal POD for both methods. It is notable that during manual scans at the frequency of 500 kHz, two of the defects were missed by two inspectors resulting in a lower $a_{90/95}$ for these scans as compared to automated ones. In manual scans, after an indication has been observed, the inspector will try to maximize the response by repeating the scan. However, this is not the case in automated scans, where the signal strength is strongly affected by both the position of the probe on the surface and the scan index. Therefore, the scan trajectory in automated scan can intercept the crack at any point across its length, which may not be necessarily the crack’s center. Considering these, the signal indications are mostly stronger in manual scans however, as mentioned earlier, some of the cracks are missed during the manual scans, leading to a better POD at 500 kHz for automated scans where all the cracks are detected. The signals recorded during automated scans are particularly weaker if the selected index is not small enough to get a response from the crack’s center. In general, it is safe to assume that the main advantage of automated scans is that no cracks are missed when small scan indexes are used. On the other hand, the signal amplitude obtained for the detected defects is normally larger in manual scans as it is best practice to maximize the response as suggested by ECT inspection procedures.

## Conclusions

- The results of the noise treatment suggest that the level of the manual noise is higher than the automated noise. This may be related to the additional gains used to compensate for the increased probe’s lift-off in manual tests. There are also some additional sources of noise which only exist in manual scans, such as variations of hand pressure and the contact between the probe and sample’s surface.
- Regardless of the chosen technique, manual or automated, the noise level increases as either the frequency is raised or the surface roughness is increased. However, the trend of these variations is steeper in manual scans. It is also observed that the variations of the noise (recorded in automated scans) versus the orientation of the tool marks on surface follow the trend of variations of $R_{a}$ itself.
- It is found that the signal amplitude of a crack whose size is smaller than the diameter of the driver’s coil is independent of the crack’s orientation. However, for cracks larger than this threshold, the difference in signal amplitude grows as the length increases. The amplitude growth rate falls to less than 5% for each orientation by reaching a certain *L/D* value at that orientation. These values are derived from simulated signals to be 1, 1.38 and 1.66 for 0°, 45°, and 90° crack orientations, respectively.
- POD of automated scans strongly depends on the selected scan index. Decreasing the index to values smaller than the driver coil’s diameter improves the POD significantly; however, the effect of further increase would be less significant.
- In general, manual scans provide better POD results than automated scans. It is believed that the tendency of inspectors to maximize the signals from the detected flaws is the main reason behind this observation. The probe’s trajectory in automated scans, even by selecting small indexes, does not always pass through the center of the crack, resulting in lower signal amplitude averages.
